# 2-*tert*-Butyl 4-methyl 3,5-dimethyl-1*H*-pyrrole-2,4-dicarboxyl­ate

**DOI:** 10.1107/S1600536812020120

**Published:** 2012-05-12

**Authors:** Hong-Xin Cai, Zhao-Po Zhang

**Affiliations:** a Department of Physics and Chemistry, Henan Polytechnic University, Jiaozuo 454000, People’s Republic of China

## Abstract

In the title mol­ecule, C_13_H_19_NO_4_, except for two C atoms of the *tert*-butyl group, the non-H atoms are almost coplanar (r.m.s. deviation = 0.2542 Å). In the crystal, mol­ecules are linked into centrosymmetric dimers by two inter­molecular N—H⋯O hydrogen bonds, forming an *R*_2_^2^(10) ring motif.

## Related literature

For complexes of Schiff bases containing a pyrrole unit, see: Wu *et al.* (2003 ▶); Wang *et al.* (2008 ▶). For the synthesis of the title compound, see: Sun *et al.* (2003 ▶).
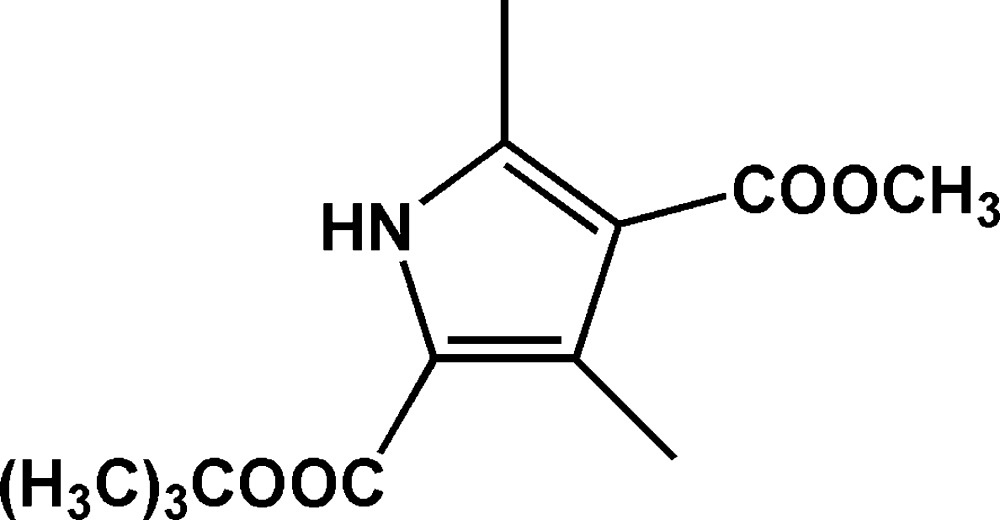


## Experimental

### 

#### Crystal data

C_13_H_19_NO_4_
*M* *_r_* = 253.29
Monoclinic, 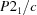

*a* = 11.788 (4) Å
*b* = 17.045 (6) Å
*c* = 7.229 (2) Å
β = 106.420 (7)°
*V* = 1393.2 (8) Å^3^
*Z* = 4
Mo *K*α radiation
μ = 0.09 mm^−1^
*T* = 296 K
0.19 × 0.18 × 0.16 mm

#### Data collection

Bruker SMART APEX CCD diffractometer
Absorption correction: multi-scan (*SADABS*; Bruker, 2007 ▶) *T*_min_ = 0.983, *T*_max_ = 0.986
7149 measured reflections
2458 independent reflections
1334 reflections with *I* > 2σ(*I*)
*R*_int_ = 0.050

#### Refinement

*R*[*F* ^2^ > 2σ(*F* ^2^)] = 0.071
*wR*(*F* ^2^) = 0.250
*S* = 1.02
2458 reflections
163 parameters
H-atom parameters constrained
Δρ_max_ = 0.28 e Å^−3^
Δρ_min_ = −0.21 e Å^−3^

### 

Data collection: *APEX2* (Bruker, 2007 ▶); cell refinement: *SAINT* (Bruker, 2007 ▶); data reduction: *SAINT*; program(s) used to solve structure: *SHELXS97* (Sheldrick, 2008 ▶); program(s) used to refine structure: *SHELXL97* (Sheldrick, 2008 ▶); molecular graphics: *SHELXTL* (Sheldrick, 2008 ▶); software used to prepare material for publication: *SHELXTL*.

## Supplementary Material

Crystal structure: contains datablock(s) I, global. DOI: 10.1107/S1600536812020120/vm2170sup1.cif

Structure factors: contains datablock(s) I. DOI: 10.1107/S1600536812020120/vm2170Isup2.hkl

Supplementary material file. DOI: 10.1107/S1600536812020120/vm2170Isup3.cml

Additional supplementary materials:  crystallographic information; 3D view; checkCIF report

## Figures and Tables

**Table 1:** Hydrogen-bond geometry (Å, °)

*D*—H⋯*A*	*D*—H	H⋯*A*	*D*⋯*A*	*D*—H⋯*A*
N1—H1*A*⋯O4^i^	0.86	2.14	2.974 (4)	165

Symmetry code: (i) 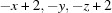.

**Fig. 1.**
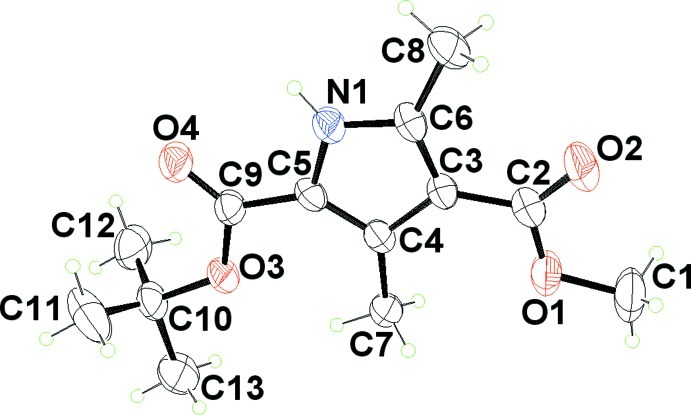
The molecular structure shown with 30% probability displacement ellipsoids.

**Fig. 2.**
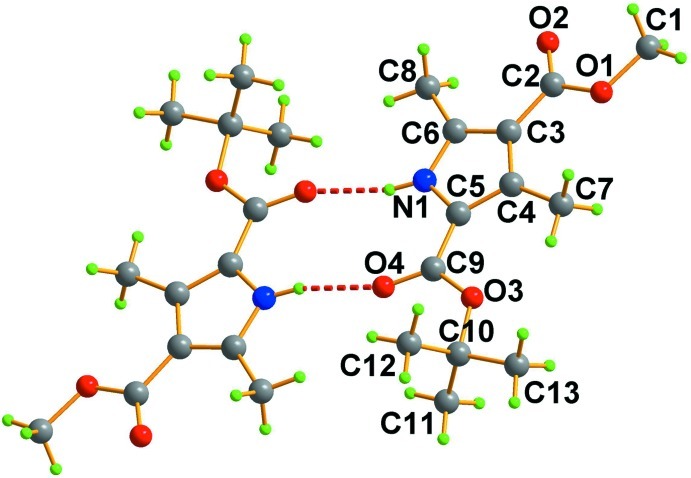
The dimer of the title compounds formed via N—H···O hydrogen bonds shown as the dashed lines. Unlabelled atoms are related with the labelled ones by symmetry operation (-x, 1/2 - y, 1/2 + z).

## supplementary crystallographic information

### Comment

Schiff bases containing pyrrole units have been extensively investigated due to their excellent coordination abilities (Wu *et al.*, 2003; Wang *et al.*, 2008). As part of our studies on bis(pyrrol-2-yl-methyleneamine) ligands, the crystal structure of the title compound is reported here.

In the title molecule (Fig. 1), except for C12 and C13 atoms of the *tert*-butyl, the non-hydrogen atoms are almost coplanar (r.m.s. deviation of the non-hydrogen atoms being 0.2542 Å). In the crystal, the molecules are linked into centrosymmetric dimers by two intermolecular N—H···O hydrogen bonds (Table 1), forming a *R*_2_^2^(10) ring motif (Fig. 2).

### Experimental

The 2-*tert*-butyl 4-methyl 3,5-dimethyl-1*H*-pyrrole-2,4-dicarboxylate was prepared by a Knorr-type reaction from the condensation of methyl acetoacetate and *tert*-butyl oximinoacetoacetate according to a literature method (Sun *et al.*, 2003).

### Refinement

The H atoms were positioned geometrically (N—H = 0.86 Å, C—H = 0.96 Å) and refined as riding with *U*_iso_(H) =1.2U_eq_(N) or 1.5U_eq_(methyl C).

### Crystal data

**Table:**

C_13_H_19_NO_4_	*F*(000) = 544
*M**_r_* = 253.29	*D*_x_ = 1.208 Mg m^−^^3^
Monoclinic, *P*2_1_/*c*	Mo *K*α radiation, λ = 0.71073 Å
Hall symbol: -P 2ybc	Cell parameters from 1135 reflections
*a* = 11.788 (4) Å	θ = 3.0–21.5°
*b* = 17.045 (6) Å	µ = 0.09 mm^−^^1^
*c* = 7.229 (2) Å	*T* = 296 K
β = 106.420 (7)°	Plate, colorless
*V* = 1393.2 (8) Å^3^	0.19 × 0.18 × 0.16 mm
*Z* = 4	

### Data collection

**Table:**

Bruker SMART CCD diffractometer	2458 independent reflections
Radiation source: fine-focus sealed tube	1334 reflections with *I* > 2σ(*I*)
Graphite monochromator	*R*_int_ = 0.050
φ and ω scans	θ_max_ = 25.0°, θ_min_ = 2.2°
Absorption correction: multi-scan (*SADABS*; Bruker, 2007)	*h* = −13→14
*T*_min_ = 0.983, *T*_max_ = 0.986	*k* = −19→20
7149 measured reflections	*l* = −8→7

### Refinement

**Table:**

Refinement on *F*^2^	Primary atom site location: structure-invariant direct methods
Least-squares matrix: full	Secondary atom site location: difference Fourier map
*R*[*F*^2^ > 2σ(*F*^2^)] = 0.071	Hydrogen site location: inferred from neighbouring sites
*wR*(*F*^2^) = 0.250	H-atom parameters constrained
*S* = 1.02	*w* = 1/[σ^2^(*F*_o_^2^) + (0.1445*P*)^2^] where *P* = (*F*_o_^2^ + 2*F*_c_^2^)/3
2458 reflections	(Δ/σ)_max_ < 0.001
163 parameters	Δρ_max_ = 0.28 e Å^−^^3^
0 restraints	Δρ_min_ = −0.21 e Å^−^^3^

### Special details

**Table:**

Geometry. All e.s.d.'s (except the e.s.d. in the dihedral angle between two l.s. planes) are estimated using the full covariance matrix. The cell e.s.d.'s are taken into account individually in the estimation of e.s.d.'s in distances, angles and torsion angles; correlations between e.s.d.'s in cell parameters are only used when they are defined by crystal symmetry. An approximate (isotropic) treatment of cell e.s.d.'s is used for estimating e.s.d.'s involving l.s. planes.
Refinement. Refinement of *F*^2^ against ALL reflections. The weighted *R*-factor *wR* and goodness of fit *S* are based on *F*^2^, conventional *R*-factors *R* are based on *F*, with *F* set to zero for negative *F*^2^. The threshold expression of *F*^2^ > σ(*F*^2^) is used only for calculating *R*-factors(gt) *etc*. and is not relevant to the choice of reflections for refinement. *R*-factors based on *F*^2^ are statistically about twice as large as those based on *F*, and *R*- factors based on ALL data will be even larger.

### Fractional atomic coordinates and isotropic or equivalent isotropic displacement parameters (Å^2^)

**Table:**

	*x*	*y*	*z*	*U*_iso_*/*U*_eq_	
C1	1.1539 (4)	0.4652 (3)	1.0373 (8)	0.1274 (19)	
H1B	1.0961	0.5063	1.0193	0.191*	
H1C	1.1953	0.4696	0.9409	0.191*	
H1D	1.2091	0.4699	1.1630	0.191*	
C2	1.1615 (3)	0.3246 (2)	1.0408 (5)	0.0768 (11)	
C3	1.0936 (3)	0.25230 (19)	1.0199 (4)	0.0587 (9)	
C4	0.9687 (3)	0.24090 (18)	0.9753 (4)	0.0554 (8)	
C5	0.9521 (2)	0.16057 (19)	0.9724 (4)	0.0586 (9)	
C6	1.1478 (3)	0.1795 (2)	1.0403 (5)	0.0657 (9)	
C7	0.8755 (3)	0.30226 (19)	0.9401 (5)	0.0692 (10)	
H7A	0.7992	0.2778	0.9138	0.104*	
H7B	0.8788	0.3337	0.8315	0.104*	
H7C	0.8881	0.3350	1.0521	0.104*	
C8	1.2746 (3)	0.1548 (3)	1.0879 (7)	0.0978 (14)	
H8A	1.2792	0.0986	1.0897	0.147*	
H8B	1.3167	0.1750	1.2123	0.147*	
H8C	1.3092	0.1750	0.9923	0.147*	
C9	0.8486 (3)	0.1108 (2)	0.9370 (5)	0.0632 (9)	
C10	0.6297 (3)	0.1201 (2)	0.8529 (6)	0.0735 (11)	
C11	0.6041 (4)	0.0676 (3)	0.6820 (8)	0.131 (2)	
H11A	0.6540	0.0222	0.7116	0.197*	
H11B	0.6189	0.0952	0.5753	0.197*	
H11C	0.5227	0.0517	0.6488	0.197*	
C12	0.6181 (4)	0.0814 (3)	1.0336 (7)	0.1063 (15)	
H12A	0.6657	0.0348	1.0584	0.159*	
H12B	0.5368	0.0677	1.0175	0.159*	
H12C	0.6443	0.1169	1.1403	0.159*	
C13	0.5522 (3)	0.1940 (3)	0.8125 (8)	0.1084 (16)	
H13A	0.5697	0.2261	0.9261	0.163*	
H13B	0.4703	0.1790	0.7776	0.163*	
H13C	0.5680	0.2229	0.7086	0.163*	
O1	1.0949 (2)	0.38906 (15)	1.0199 (4)	0.0925 (9)	
O2	1.2657 (3)	0.32986 (18)	1.0728 (6)	0.1295 (13)	
O3	0.74888 (17)	0.15373 (14)	0.8891 (3)	0.0713 (8)	
O4	0.85058 (19)	0.03974 (15)	0.9488 (4)	0.0876 (9)	
N1	1.0615 (2)	0.12471 (17)	1.0127 (4)	0.0674 (8)	
H1A	1.0730	0.0748	1.0192	0.081*	

### Atomic displacement parameters (Å^2^)

**Table:**

	*U*^11^	*U*^22^	*U*^33^	*U*^12^	*U*^13^	*U*^23^
C1	0.101 (3)	0.078 (3)	0.197 (6)	−0.040 (3)	0.032 (3)	−0.010 (3)
C2	0.053 (2)	0.083 (3)	0.088 (3)	−0.0142 (19)	0.0105 (18)	0.0009 (19)
C3	0.0448 (18)	0.064 (2)	0.066 (2)	−0.0079 (15)	0.0136 (14)	0.0011 (15)
C4	0.0457 (17)	0.063 (2)	0.0539 (18)	−0.0049 (14)	0.0090 (13)	−0.0018 (14)
C5	0.0379 (16)	0.070 (2)	0.064 (2)	−0.0014 (14)	0.0084 (13)	−0.0017 (16)
C6	0.0442 (18)	0.078 (2)	0.074 (2)	−0.0119 (16)	0.0149 (15)	−0.0050 (16)
C7	0.0512 (19)	0.060 (2)	0.091 (2)	0.0005 (16)	0.0119 (17)	0.0041 (17)
C8	0.043 (2)	0.099 (3)	0.146 (4)	0.005 (2)	0.019 (2)	−0.001 (3)
C9	0.0475 (19)	0.058 (2)	0.083 (2)	−0.0048 (15)	0.0169 (16)	−0.0033 (17)
C10	0.0416 (18)	0.074 (2)	0.102 (3)	−0.0111 (16)	0.0146 (17)	−0.009 (2)
C11	0.067 (3)	0.164 (5)	0.141 (4)	−0.010 (3)	−0.005 (3)	−0.062 (4)
C12	0.075 (3)	0.098 (3)	0.152 (4)	−0.020 (2)	0.041 (3)	0.009 (3)
C13	0.050 (2)	0.110 (4)	0.159 (4)	0.008 (2)	0.018 (2)	0.002 (3)
O1	0.0673 (17)	0.0635 (16)	0.145 (3)	−0.0193 (14)	0.0268 (16)	−0.0068 (15)
O2	0.0530 (18)	0.103 (2)	0.215 (4)	−0.0239 (15)	0.0100 (19)	0.007 (2)
O3	0.0359 (12)	0.0687 (15)	0.1046 (19)	−0.0050 (10)	0.0120 (11)	0.0016 (12)
O4	0.0523 (14)	0.0607 (17)	0.144 (2)	−0.0030 (11)	0.0180 (14)	−0.0099 (14)
N1	0.0442 (15)	0.0624 (16)	0.093 (2)	−0.0020 (13)	0.0149 (13)	−0.0030 (14)

### Geometric parameters (Å, º)

**Table:**

C1—O1	1.461 (5)	C8—H8B	0.9600
C1—H1B	0.9600	C8—H8C	0.9600
C1—H1C	0.9600	C9—O4	1.215 (4)
C1—H1D	0.9600	C9—O3	1.344 (4)
C2—O2	1.188 (4)	C10—O3	1.470 (4)
C2—O1	1.334 (4)	C10—C11	1.486 (6)
C2—C3	1.454 (5)	C10—C12	1.504 (6)
C3—C6	1.385 (5)	C10—C13	1.534 (5)
C3—C4	1.429 (4)	C11—H11A	0.9600
C4—C5	1.383 (4)	C11—H11B	0.9600
C4—C7	1.486 (4)	C11—H11C	0.9600
C5—N1	1.382 (4)	C12—H12A	0.9600
C5—C9	1.448 (4)	C12—H12B	0.9600
C6—N1	1.354 (4)	C12—H12C	0.9600
C6—C8	1.496 (5)	C13—H13A	0.9600
C7—H7A	0.9600	C13—H13B	0.9600
C7—H7B	0.9600	C13—H13C	0.9600
C7—H7C	0.9600	N1—H1A	0.8600
C8—H8A	0.9600		
			
O1—C1—H1B	109.5	O4—C9—O3	124.0 (3)
O1—C1—H1C	109.5	O4—C9—C5	125.0 (3)
H1B—C1—H1C	109.5	O3—C9—C5	111.0 (3)
O1—C1—H1D	109.5	O3—C10—C11	110.0 (3)
H1B—C1—H1D	109.5	O3—C10—C12	109.5 (3)
H1C—C1—H1D	109.5	C11—C10—C12	114.2 (4)
O2—C2—O1	120.2 (4)	O3—C10—C13	101.7 (3)
O2—C2—C3	126.4 (4)	C11—C10—C13	111.4 (4)
O1—C2—C3	113.4 (3)	C12—C10—C13	109.3 (3)
C6—C3—C4	108.5 (3)	C10—C11—H11A	109.5
C6—C3—C2	121.7 (3)	C10—C11—H11B	109.5
C4—C3—C2	129.8 (3)	H11A—C11—H11B	109.5
C5—C4—C3	105.7 (3)	C10—C11—H11C	109.5
C5—C4—C7	126.9 (3)	H11A—C11—H11C	109.5
C3—C4—C7	127.4 (3)	H11B—C11—H11C	109.5
N1—C5—C4	108.4 (3)	C10—C12—H12A	109.5
N1—C5—C9	117.9 (3)	C10—C12—H12B	109.5
C4—C5—C9	133.7 (3)	H12A—C12—H12B	109.5
N1—C6—C3	107.3 (3)	C10—C12—H12C	109.5
N1—C6—C8	120.0 (3)	H12A—C12—H12C	109.5
C3—C6—C8	132.6 (3)	H12B—C12—H12C	109.5
C4—C7—H7A	109.5	C10—C13—H13A	109.5
C4—C7—H7B	109.5	C10—C13—H13B	109.5
H7A—C7—H7B	109.5	H13A—C13—H13B	109.5
C4—C7—H7C	109.5	C10—C13—H13C	109.5
H7A—C7—H7C	109.5	H13A—C13—H13C	109.5
H7B—C7—H7C	109.5	H13B—C13—H13C	109.5
C6—C8—H8A	109.5	C2—O1—C1	118.1 (3)
C6—C8—H8B	109.5	C9—O3—C10	123.8 (3)
H8A—C8—H8B	109.5	C6—N1—C5	110.1 (3)
C6—C8—H8C	109.5	C6—N1—H1A	124.9
H8A—C8—H8C	109.5	C5—N1—H1A	124.9
H8B—C8—H8C	109.5		
			
O2—C2—C3—C6	1.3 (6)	N1—C5—C9—O4	3.7 (5)
O1—C2—C3—C6	−178.8 (3)	C4—C5—C9—O4	−176.5 (4)
O2—C2—C3—C4	−177.7 (4)	N1—C5—C9—O3	−176.4 (3)
O1—C2—C3—C4	2.2 (5)	C4—C5—C9—O3	3.4 (5)
C6—C3—C4—C5	0.6 (4)	O2—C2—O1—C1	0.1 (6)
C2—C3—C4—C5	179.7 (3)	C3—C2—O1—C1	−179.8 (3)
C6—C3—C4—C7	−179.6 (3)	O4—C9—O3—C10	2.7 (5)
C2—C3—C4—C7	−0.6 (6)	C5—C9—O3—C10	−177.2 (3)
C3—C4—C5—N1	−0.2 (3)	C11—C10—O3—C9	−63.7 (5)
C7—C4—C5—N1	−179.9 (3)	C12—C10—O3—C9	62.6 (4)
C3—C4—C5—C9	180.0 (3)	C13—C10—O3—C9	178.1 (3)
C7—C4—C5—C9	0.2 (6)	C3—C6—N1—C5	0.7 (4)
C4—C3—C6—N1	−0.8 (4)	C8—C6—N1—C5	179.4 (3)
C2—C3—C6—N1	−180.0 (3)	C4—C5—N1—C6	−0.4 (4)
C4—C3—C6—C8	−179.3 (4)	C9—C5—N1—C6	179.5 (3)
C2—C3—C6—C8	1.5 (6)		

### Hydrogen-bond geometry (Å, º)

**Table:**

*D*—H···*A*	*D*—H	H···*A*	*D*···*A*	*D*—H···*A*
N1—H1*A*···O4^i^	0.86	2.14	2.974 (4)	165

Symmetry code: (i) −*x*+2, −*y*, −*z*+2.
